# Strategies for modeling aging and age-related diseases

**DOI:** 10.1038/s41514-024-00161-5

**Published:** 2024-07-10

**Authors:** D. Jothi, Linda Anna Michelle Kulka

**Affiliations:** 1 Department of Biochemistry II, Friedrich Schiller University, Jena, Germany; 2 Institute for Physiological Chemistry, Martin Luther University Halle-Wittenberg, Halle, Germany

**Keywords:** Induced pluripotent stem cells, Ageing, Ageing

## Abstract

The ability to reprogram patient-derived-somatic cells to IPSCs (Induced Pluripotent Stem Cells) has led to a better understanding of aging and age-related diseases like Parkinson’s, and Alzheimer’s. The established patient-derived disease models mimic disease pathology and can be used to design drugs for aging and age-related diseases. However, the age and genetic mutations of the donor cells, the employed reprogramming, and the differentiation protocol might often pose challenges in establishing an appropriate disease model. In this review, we will focus on the various strategies for the successful reprogramming and differentiation of patient-derived cells to disease models for aging and age-related diseases, emphasizing the accuracy in the recapitulation of disease pathology and ways to overcome the limitations of its potential application in cell replacement therapy and drug development.

## Introduction

Aging is a complex phenomenon influenced by both genetic and environmental factors. An increase in the rate of biological aging in humans is often associated with the development of age-related neurodegenerative disorders like Alzheimer’s (AD) and Parkinson’s (PD)^1,2^. To date, various cell and animal models have been exploited to understand the mechanism of aging and age-related diseases, and some of them are discussed below.

Aging in animals and especially in humans is the natural result of entropy in cells as part of tissues and organs of the human body. It is a slow, inexorable process that leads to a continuous increase in molecular errors in DNA and proteins, increasingly disrupting the homeostasis of cells, tissues, and organs^3,4^. Accordingly, a set of tools has been established to analyze aging on a cellular level. Cellular aging is a representation of replicative senescence, a process by which somatic cells lose their proliferative capacity and stop dividing following a cell cycle arrest. In vitro, human fibroblast cells are used to study the process of replicative senescence because of their limited proliferative capacity. Senescence in these cells is assessed by an increase in β-galactosidase activity or expression of cell cycle restricting tumor suppressor proteins such as p14^ARF^, p16, p21, and p53^5,6^. Alternatively, cellular aging can also be characterized by the expression of aging markers such as IGF1, EGF, and c-Fos in cells^7^. In addition, the measurement of the population doubling of cells allows quantification of the number of cells associated with replicative aging^8^. However, studying replicative senescence is often time-consuming, as numerous population doublings are required before cells finally acquire senescence characteristics. In addition, various factors can induce stress, thus affecting cell culture conditions and accelerating senescence. This raises the question of whether the changes we observe as replicative senescence in cells are an accurate representation of senescence in vivo. Nevertheless, it is crucial to acknowledge that traditional cell culture methods have led to some basic understanding of aging hallmarks such as telomere attrition and genomic instability^9,10^.

Because primary cell models, such as human fibroblasts, cannot accurately mimic in vivo conditions, various animal models have been exploited to provide important information about aging and age-related diseases. Many conserved pathways that regulate the lifespan and health span of organisms have been identified in these model organisms and several medical interventions have been proposed so far. Interestingly, the calorie restriction (CR) strategy administered with a standard diet extended most model organisms’ lifespans^11–13^. However, the overall mechanism by which CR extends the lifespan in these organisms isn’t clear. Nevertheless, implementing model organisms to study aging has its own limitations. Most of the interventions developed based on the animal models are sex and strain-specific. In fact, despite similar genetic backgrounds, variation in mean lifespan accounted for up to 20% due to varying sex and strain of the mouse^14^. Moreover, mice and rat models do not exhibit all the disease phenotypes of AD and PD. Although long-living naked mole rats displayed signs of aging consistent with humans such as retinal degeneration and osteoarthritis, they exhibited no change in senescence and age-related mortality with age^15^. However, research on aging using model organisms is essential as human subjects have ethical and moral limits.

One promising possibility to study cellular aging in human cells is offered by induced pluripotent stem cells (IPSCs). The advent of IPSC technology made it possible to generate multiple isogenic cell types with similar genotypes. The differentiation of brain cells from IPSCs and the development of co-culture platforms offer valuable insights into cell type-related dysfunction in aging, AD, and PD. Moreover, using the co-culture system, it’s now possible to study the interaction of neuronal and non-neuronal brain cells like astrocytes, microglia, oligodendrocytes, endothelial cells, and pericytes. Notably, IPSCs derived from familial AD (FAD) and late-onset Alzheimer’s disease (LOAD) patients displayed disease-related features that correlate with the prodromal changes towards the development of AD in patients^16–19^. Moreover, IPSCs generated from a subject carrying the specific genetic mutation could facilitate patient-tailored treatments. 3D models of IPSCs mimicking the blood-brain barrier (BBB) would facilitate the discovery of systematic drugs that can enter the brain and verify if BBB dysfunction is linked to AD and PD^20^. Besides, neuron-based 3D models were developed by incorporating IPSC-derived microglia of FAD patients into organoids of FAD patients carrying APOE ε3- and APOE ε4-containing cells. This led to the finding that APOE ε3 microglia exhibited less amyloid-β (Aβ) than APOE ε4 microglia^16,21^. Furthermore, an artificial heart tissue model developed using IPSCs for investigating aging in cardiac muscle, observed that with advancing age, progressive degeneration occurs, including structural changes such as stiffness as well as functional decline^22^. This was manifested by an increase in cardiac markers, reduced expression of the proliferation marker ki67, and an increase in the CDK inhibitor p21. A model for blood vessels consisting of vascular networks derived from iPSCs was developed for examining their interactions with nanomaterials^23^. It was found that the toxicity profile of the nanoparticles for iPSC-derived endothelial cells vary depending on both the age of the endothelial cells and the format of the vascular network. Cerebral organoids were developed exhibiting features of Ataxia-Telangiectasia, a genetic disorder associated with premature aging and other health issues^24^. This led to the finding that the activation of the cGAS-STING pathway, a part of the immune response, leads to premature cellular aging. Furthermore, cerebral organoids derived from iPSCs irradiated with gamma radiation to induce cell damage followed by the investigation of whether anti-aging substances minocycline and rapamycin play a role in cell viability, proliferation, and differentiation^25^. It was identified that the pre-conditioning of neurospheres to rapamycin before irradiation and to minocycline after irradiation confers neuroprotection and rescues developmental potential of the cerebral organoid. Moreover, Omics analysis of IPSC-derived neurons and brain tissue of 53 individuals (16 with clinical and pathological profile of AD) revealed that the key neuropathological features such as amyloid-β and p-tau accumulation of IPSC-derived neurons correlated with the levels of plaque and p-tau in the brain tissue of the same individual^26^. Thus, IPSC-based 3D models are a powerful tool to analyze aging, disease-related phenotypes and to perform drug screening. Though developing more complex 3D organoid brain models with mature brain cells is a work in progress, the improvisation of these models will be of great utility in the future.

This review mainly discusses strategies for in vitro models of aging and age-related diseases such as AD and PD, and their potential applications in drug development and regenerative medicine, highlighting the accuracy of disease pathology recapitulations.

## Modelling aging and age-related diseases

### Aging-induced model

Several 2D and 3D culture models have been developed to manifest aging phenotype in rejuvenated IPSCs^27^. Although majority of the IPSC-based aging models are 2D, recent research on tissue engineered 3D models offer valuable information on cell-cell and cell-ECM interactions relevant to the mechanism of aging^28,29^.

#### Strategies for inducing aging phenotype in IPSC-derived cells

Reprogramming resets the aging phenotype, resulting in the loss of age-associated markers in IPSCs of aged donors^30–32^. Hutchinson-Gilford progeria syndrome (HGPS)-iPSCs, upon differentiation, expressed age-associated markers such as DNA damage and an increase in mitochondrial ROS (Mito-ROS). This indicates that genetic mutations in LMNA, resulting in abnormal production of progerin, could be causative^33^. Following this observation, progerin overexpression was used as a strategy to induce aging in iPSCs, irrespective of age. The resulting mDA neurons showed dendrite degeneration, formation of inclusion bodies, reduced TH+ (Tyrosine hydroxylase) neurons, accumulation of DNA damage and Mito-ROS but no changes in senescence, suggesting that the strategy doesn’t completely recapitulate the aging phenotype in mDA neurons. Interestingly, when the similar strategy is used in iPSCs differentiated to fibroblasts, the resulting fibroblasts displayed increase in DNA damage, Mito-ROS, senescence and a decrease in telomere length replicating key aging features effectively.

Aging can also be readily induced in cells through the long-term culture of IPSC-derived cells^22,34,35^. For instance, induced cardiomyocytes (iCMs) derived from human IPSC exhibit peak maturation by day 55 of differentiation culture followed by functional deterioration and arrival of aging markers like accelerated senescence, increase in p21 expression, and presence of lipofuscin granules by day 120^22,36,37^. Additionally, engineered 3D tissue models with aged iCMs (generated by long-term culture), possessing stiffness comparable to that of the aged human heart, demonstrate lower survival rates in response to the stress of 48 h hypoxia followed by 24 h normoxia, less proliferative capacity, higher β-gal activity, lipofuscin accumulation, lower cardiac beating velocity, and higher ROS levels compared to young tissue models^22,38^.

However, when aged iCMs induced by the long-term strategy were placed on the young cardiac extra-cellular matrix (ECM) from mice aged 1-3 months, they rejuvenated. This indicates that some of the aged iCMs were in a quiescent state rather than a senescent state, suggesting that not all aged cells were effectively driven into senescence by the long-term culture strategy^22,34,38^. On the other hand, young iCMs (1–2 months old) seeded onto aged mice ECM (22–24 months old) showed enhanced cardiac beating velocity, 8% reduction in Ki67 proliferation marker, larger lipofuscin accumulation area, and longer sarcomeres indicating iCMs cultured on aged ECMs better replicate aging phenotype.

Furthermore, aging phenotype can be induced by exposure to ROS inducing agents, ionizing radiation and by shortening telomeres of human IPSC-derived lineages^39–42^. Cerebral organoid cultured under hypoxic conditions exhibited BBB dysfunction, increased oxidative stress, and elevated secretion of inflammatory cytokines such as IL-1β, TNF-α and IL-6^41^. Moreover, Organoids irradiated with 0.5 or 2 Gy of 250 MeV protons for 30 mins, 24 h, and 48 h exhibited time and dose-dependent increase in DNA damage respectively^42^. Pharmacological inhibition of telomerase activity by 10 μM telomerase inhibitor BIBR1532 increases the percentage of short telomeres by 4.8% in PINK1 and 2.12% in PARKIN mutant-derived midbrain dopaminergic (mDA) neuron at differentiation day 65. Moreover, aging features such as increased DNA damage, increased mitochondrial superoxide, and loss of the midbrain dopaminergic (mDA) neuron marker TH are observed in both PINK1, and PARKIN mutant-derived neuronal cells at differentiation day 65^39^. This suggest that the strategy renders advantage of altering both aging hallmarks and specific neuronal aging markers and would be beneficial for studying whether the regulation of general aging hallmarks precedes neuronal aging in this case. Pharmacological inhibition of hIPSC has its limitations such as decrease in the cell surface marker SSEA3, and reduction in proliferating cells. This could be eliminated alternatively by using CRISPR-based genetic manipulation^40^. CRISPR-based knock-out of the TERT gene in iPSCs displayed aging features such as increased DNA damage, inflammation, and senescence in both differentiated motor neurons and astrocytes. Additionally, motor neurons exhibited fewer neurites and decreased soma size, while astrocytes showed an increased cell area and elevated levels of glial fibrillary acidic protein, typical characteristic of neuronal and astrocyte aging.

The optimal approach would be to combine 3D tissue model with genetic manipulation using CRISPR technology. For example, knockout of Hypoxia-inducible factor 1α (HIF-1 α) in 3D vascular tissues derived from hIPSC resulted in the tissue impairment such as decrease in 3D lumen formation and increase in mito ROS, decrease in 2D tube formation and decreased survival of endothelial cells under ischemic treatment^43^. Spinal cord organoids generated by TERT low neural progenitor cells (NPCs) in matrigel, were smaller in size compared to the control organoids. Additionally, TERT low organoids revealed a decrease in proliferation, increase in cell death, and loss of developmental signatures like neural rosette structures^40^. Combining a 3D tissue model with a genetic editing strategy offers a dual benefit, allowing the examination of the impact of both cell-ECM and specific mutation on the aging phenotype. Alternatively, 3D aging models can also be directly developed from the cells with premature aging genotype eliminating the need for genetic alterations^24^. Tissue engineered blood vessel model of HGPS from IPSCs of endothelial cells and smooth muscle cells displayed a reduction in vasodilation and vasoconstriction^44^. However, when 3D tissue model is generated with HGPS-IPSCs-derived endothelial cells and healthy smooth muscle cells, only reduction in vasodilation phenotype was observed, suggesting the crucial role of endothelial cells in HGPS disease development.

### Werner syndrome-based model

Werner syndrome (WS) is a rare genetic disorder that causes premature aging in humans due to telomere dysfunction. Patients generally carry two copies of the defective gene WRN (maintains the DNA integrity) and inherit the disease in an autosomal recessive pattern^45^. Premature aging begins in early adulthood with an increased risk of cataracts, osteoporosis, type 2 diabetes, skin ulcers, and cancer. Individuals with this syndrome generally have a shorter lifespan and live until their mid-40s^46^. The treatment includes managing disease symptoms; no promising therapy has been developed so far. Thus, the generation of IPSC models from WS patients could be a promising tool for drug development and deciphering the mechanism of the disease progression.

#### Strategies for modelling Werner syndrome-based cells

**Fig. 1:**
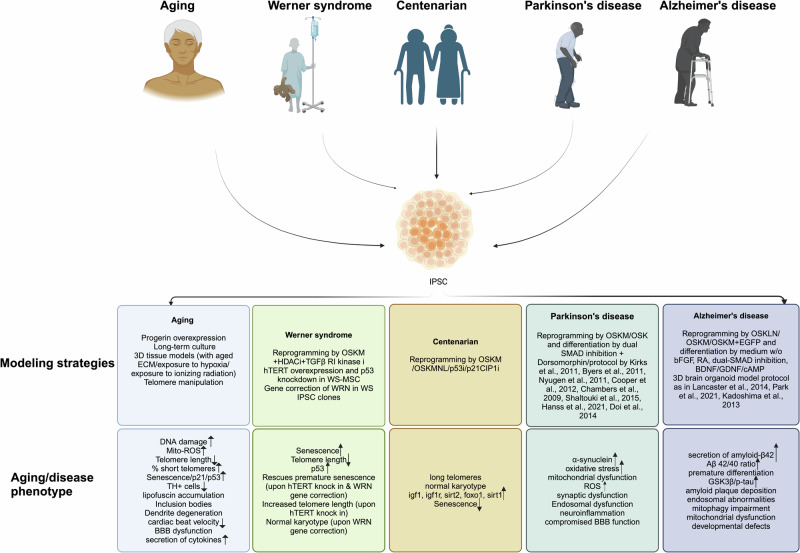
Schema depicting the summary of modeling strategies for patient-derived cells, and associated aging/disease phenotype recapitulation of aging and age-associated diseases (AD & PD) as presented in Table 1. “↑” represents increase and “↓” represents decrease. Schema generated in Bio-render.

The successful reprogramming of IPSCs from WS fibroblasts was first reported in 2014^47^. WS-fibroblasts generally exhibit a slow rate of division, and rapid transition into a senescent state, a hallmark of premature aging. WS fibroblasts were reprogrammed using HDAC inhibitor and TGFβ RI kinase inhibitor in addition to the transduction of OSKM (Oct4, Sox2, Klf4, c-Myc) factors (Fig. 1). Unlike WS fibroblasts, WS-derived IPSCs displayed long telomeres and chromosomal stability. These IPSCs also exhibited appropriate pluripotency markers and were morphologically similar to hESCs^48^. In addition, the differentially expressed genes in WS fibroblasts to WS IPSC were reduced from 858 to 27 after reprogramming resetting the differential expression of aging-associated genes. However, minor limitations such as a slow rate of DNA synthesis and a slight deficit in telomere synthesis were observed in WS-IPSCs with this protocol.

Unexpectedly, premature senescence reoccurred when WS-IPSCs were differentiated into WS-MSC (Mesenchymal Stem Cells) but not in WS-NPCs (neural progenitor stem cells). WS-MSCs exhibited a lower population doubling, a higher number of cells stained positive for senescence-associated β-gal activity, reduced telomere length, and higher p53 expression compared to normal MSC and WS-NPC. This indicates that there is lineage-specific protection of the differentiated cells from premature aging. Likewise, WS keratinocytes are also reported to be protected from premature senescence by telomere maintenance^49^. The reason behind lineage-specific premature senescence in WS-MSC is the loss of telomerase activity and higher expression of p53. Hence, upon hTERT knock-in, reprogramming of WS fibroblasts resulted in the WS IPSCs with high telomerase activity and telomere length. In line with this, exogenous expression of hTERT is reported to enhance the reprogramming ability of the homozygous mutant form of WS fibroblasts which otherwise could not be successfully reprogrammed by the 4-factor method^50^. After subsequent differentiation, the telomerase activity of hTERT knock-in WS-MSC cells remains intact. Moreover, the knock-in of hTERT in WS MSCs with WRN deficit background rescued the senescence-like phenotype by downregulating p16 and DNA damage marker γH2AX. Alternatively, WS cells enter senescence via the p53 pathway irrespective of the activity of the telomerase suggesting p53 runs downstream of the hTERT in the senescence regulation^51^. Hence, p53 inhibition in WS fibroblasts rescues senescence-like phenotype in WS-MSC without the necessity of telomere resetting. Nonetheless, the use of the p53i strategy may lead to an elevated risk of tumorigenesis and genomic instability. Consequently, hTERT overexpression appears to offer a favourable alternative to the p53i approach for rescuing premature senescence in WS-MSCs with WRN deficit background.

Alternatively, to address lineage-specific premature aging that prevails in WS-MSCs, gene correction of WS cells can be employed to ameliorate the senescence phenotype. The point mutation C > T in exon 9 resulting in the truncated WRN expression can be corrected by CRISPR/Cas9 technology in IPSC clones. Following the differentiation of these clones, WS-MSCs showed a higher proliferation rate, and no premature senescence relative to uncorrected WS-MSCs^52^. While gene correction of WS-IPSCs aims to eradicate premature senescence in WS-MSCs, alternative approaches such as hTERT overexpression or p53 inhibition prove to be more cost-effective and efficient.

On the other hand, gene correction of WS IPSC clones (*WRN*: c.3139-1 G > C) eliminates the need for HDACi and TGFβ RI kinase I for the successful generation of WS-IPSCs. Peripheral blood mononuclear cells from WS patients harbouring a homozygous mutation of *WRN*: c.3139-1 G > C were successfully reprogrammed by introducing OSKM through the Sendai-virus system^53^. Following gene correction by CRISPR/Cas9-mediated technology, the resulting IPSC clones exhibited IPSC-like morphology, higher expression of pluripotent factors, normal karyotype, and ability to differentiate into three germ layers. This suggests a possible clinical application of WS IPSCs. Nevertheless, such applications require a careful evaluation of WS IPSC clones, and screening for genetic, and epigenetic changes.

### Centenarian-based model

Gerontologists define centenarians as individuals who attain the age of 100 or beyond, while supercentenarians are those who reach 110 years of age or even exceed it^54^. A multitude of investigations have demonstrated that an array of factors, encompassing lifestyle, genetics, surroundings, and character traits, collectively contribute to the extended lifespans observed in centenarians and supercentenarians^55–59^. Research conducted on supercentenarians from Okinawa disclosed that many of them had experienced fewer instances of illness before reaching 100 years of age^59^. This underscores the notion that centenarians and supercentenarians possess a distinct advantage conducive to their long-term survival.

Apart from the common genetic and epigenetic barriers of reprogramming^60,61^, IPSC generation from centenarians poses additional challenges as the probability of successful reprogramming decreases with the increase of donor age^62^. For example, IPSC colony formation in 23-month-old mice required the treatment of 4-factor twice compared to 2-month-old mice and yet delayed IPSC colony formation by 15 days^63^. The challenges are often associated with cell-intrinsic properties such as higher incidence of senescence as the expression of senescence markers like p21^CIP1^, and p16^INK4A^ increase with age^64^, accumulation of age-associated genetic mutation, and retention of age-related epigenetic signature^65^. Surprisingly, the strong linear relationship between age and somatic mutations seems to decline after 90 years of age suggesting that extremely elderly individuals (>90 years of age) harbour a protective mechanism against the aging-associated somatic mutation^65^.

#### Strategies for modelling centenarian-based cells

B-lymphoblastoid cell lines (LCL) of a rare supercentenarians’ (SC) population aged 114 were successfully reprogrammed using the 4-factor method OSKM (Oct4, Sox2, Klf4, c-Myc)^66,67^ with the efficiency of 0.01%. The resulting SC-IPSC lines exhibited pluripotent properties, long telomeres, a normal karyotype, and the ability to differentiate into three germ layers, which is similar to IPSC lines derived from healthy donors^68^. Whole transcriptome analysis showed that the gene clusters of SC-LCL were distinguishable from young and old-aged LCL before reprogramming. However, after reprogramming, only 0.08% of differential expression was identifiable, indicating that most of the differential expression of genes was eliminated^66^. Nonetheless, only one out of three clones were successfully reprogrammed in this study, as the other two clones exhibited a lower incidence of telomere resetting with the shortest average telomere lengths of around 10 kbp. In addition, SC-IPSC clones exhibited a higher percentage of critically short telomeres of length <3 kbp. This suggests possible limitations associated with telomere resetting in reprogramming from SC-LCL which require further investigation.

Another group of researchers^69^ reported having successfully reprogrammed dermal fibroblasts from centenarians aged 106,109 using the conventional 4-factor (OSKM) method (Fig. 1). The resulting IPSC clones displayed similar morphological characteristics to embryonic stem cells and could differentiate into different cell lineages, as confirmed by teratoma formation. Additionally, the telomere length of the IPSC clones was 1.3 times greater than that of the original fibroblasts. Transcriptome analysis indicated that there were no significant differences in the expression profile of certain longevity genes, such as igf1, igf1r, sirt2, foxo1, and sirt1, between the centenarian IPSC clones and the IPSC line 201B7 (control) derived from a Caucasian family. However, it is important to note that this study only examined a few longevity genes, missing out on some of the major longevity genes like APOE, p53, and KLOTHO^70^. Furthermore, this research did not consider the influence of age-related epigenetic memory on C-IPSCs and lacked comprehensive information regarding the efficiency of generating IPSC clones from the centenarians.

An alternative strategy of reprogramming fibroblasts using the 6-factor combination OSKMNL (Oct4, Sox2, Klf4, c-Myc, Nanog, Lin28) was proposed^31^ for the elderly (aged 92, 94, 96, 101) to overcome the senescent-associated reprogramming barriers. The addition of NANOG initiates reprogramming independently of the cell division rate, while the presence of the LIN28 factor enhances the division rate of somatic cells, ultimately yielding IPSC lines that closely mimic the characteristics of hESCs. Transcriptome analysis showed that the gene expression profile of the 6-factor IPSCs was reset to an embryonic-like pluripotent state. Moreover, some IPSC clones generated by the 6-factor method had telomeres longer than the hESCs with a length above 21.2 kbp, and the expression of hTERT was almost 5 times greater than that of hESCs indicating the method’s greater efficiency in telomere resetting. Notably, the reprogramming efficiency of 6-factor OSKMNL is 0.06% which is quite greater than that of the reprogramming efficiency of the 4-factor method (0.01%) employed for reprogramming supercentenarians from B-LCL^66^. The study also observed that the promoter regions of OCT4 and NANOG were demethylated after successful reprogramming, which is known to induce pluripotency and enhance self-renewal^71^. The physiological changes associated with aging were reversed upon redifferentiation, and the re-differentiated fibroblasts exhibited similar characteristics to young embryonic fibroblasts. This suggests its potential applications in cell-based therapies for elderly patients. However, the research did not broadly investigate the methylation patterns of cancer-related genes such as TEAD3, ADGRL4, and AHRR, which typically remain methylated due to defects in demethylation during the reprogramming process^65^.

Although generating IPSCs from extremely elderly individuals can be challenging due to the senescence-associated barriers, several approaches can overcome these limitations. These include inhibiting p53 and p21^CIP1^ ^64^, using additional reprogramming factors such as NANOG and LIN28, or reprogramming using progenitor cells/stem cells as a starting cell. These strategies can enhance the potential applications of IPSCs from centenarians. Centenarian-IPSCs can serve as a control for healthy individuals for comparison with elderly diseased individuals providing insights into how centenarians managed to remain healthy as they age. While there are concerns about the clinical application of IPSCs from elderly individuals due to the risk of transfer of age-associated somatic mutation, the development of improvised reprogramming strategies that can reset genetic and epigenetic barriers may alter this perspective in the future.

### Neurodegenerative-based model

#### Alzheimer’s

Alzheimer’s is one of the most common neurodegenerative disorders characterized by the accumulation of Aβ (amyloid-β) and tau protein^72,73^. Familial Alzheimer’s disease (FAD) is a rare genetic form of Alzheimer’s disease that is accompanied by inherited mutations in one of three genes: amyloid precursor protein (APP), presenilin 1 (PSEN1), or presenilin 2 (PSEN2)^74,75^. These mutations result in the accumulation of amyloid-β protein in the brain of Alzheimer’s patients. FAD accounts for less than 5% of all Alzheimer’s cases and tends to develop earlier in life, usually before age 60. Sporadic Alzheimer’s disease (SAD) is the most common form of Alzheimer’s, accounting for about 95% of all AD cases^75^. SAD typically develops later in life, usually after the age of 65, and is not caused by genetic mutations in most cases, however, there is increasing evidence that the genetic variant of APOE ε is associated with LOAD^21,76^. While the exact cause of SAD is not known, it is believed to be the result of a complex interplay of genetic, environmental, and lifestyle factors that contribute to the development of the disease. Both FAD and SAD share symptoms, including memory loss, difficulty with language, and problems with cognitive function. However, FAD tends to progress more rapidly than SAD and is more likely to be accompanied by other neurological symptoms, such as seizures and myoclonus (sudden muscle jerks)^77,78^.

Besides neuronal, non-neuronal cells, such as astrocytes and microglia, also play an important role in Alzheimer’s disease. These cells are collectively referred to as glial cells and are essential for the proper functioning of the nervous system. In Alzheimer’s disease, astrocytes can become activated in response to the accumulation of amyloid-β protein and tau protein and contribute to the progression of the disease by secretion of pro-inflammatory cytokines, reactive oxygen species, and other molecules that can damage neurons^79,80^. Microglia are immune cells in the brain that function as the first line of defence against injury and infection. In Alzheimer’s disease, microglia can become activated and release pro-inflammatory molecules, leading to inflammation and further damage to neurons^81,82^. Overall, the role of non-neuronal cells in Alzheimer’s disease is complex and not yet fully understood, but research suggests that targeting these cells could be a potential therapeutic approach for treating the disease.

#### Strategies for modelling Alzheimer’s

Several approaches have been developed to model Alzheimer’s using IPSCs (Table 1). The first IPSC clones generated from FAD patients with PSEN1 (A246E) and PSEN2 (N141l) mutations were obtained by reprogramming using five factors such as OSKLN (OCT4, SOX2, KLF4, LIN28, and NANOG)^83^ (Fig. 1). Following differentiation in hIPSC medium without bFGF, neurons displayed higher secretion of amyloid-β42 reaching 80 pmol/l, in contrast to the control group, which had levels around 15 pmol/l. However, no accumulation of tau protein was observed during the 2-week culture period of neurons suggesting that the model failed to mimic the tau pathology with the current protocol. A similar attempt to derive neurons from PSEN1 mutant A246E fibroblasts through reprogramming by OSKM and differentiation by dual SMAD inhibition resulted in the neurons exhibiting increase in Aβ 42/40 ratio, mitophagy impairment, and dysfunctional mitochondria^84,85^.

**Table 1:** Detailed description of reprogramming, differentiation strategies, resulting aging/disease phenotype, and relevant reports summarized

Aging/Age-related diseases	Key phenotypic markers	Mutation type	Differentiation process	Strategy	Aging/disease phenotype	Reports
Aging induced model	DNA damage Increase in Mito-ROS Increase in senescence/ p21/ β-gal activity lipofuscin accumulation Telomere shortening	Null	Old/young donor IPSC --> fibroblasts	Progerin overexpression in IPSC-derived fibroblasts	**Fibroblasts displayed:** DNA damage Increase in Mito-ROS Decrease in telomere length Increase in % short telomeres Increase in senescence	Miller et al., 2013
		Null	Old/young donor IPSC --> mDA neurons	Progerin overexpression in IPSC-derived mDA neurons	**mDA neurons displayed:** DNA damage Increase in Mito-ROS Dendrite degeneration Inclusion bodies Reduced TH+ neurons	Miller et al., 2013
		Null	IPSC --> cardiomyocyte	Long-term culture of IPSC-derived cells	**Induced cardiomyocytes displayed:** Accelerated senescence Increase in p21 and p53 lipofuscin accumulation	Acun et al., 2019
		Null	IPSC --> cardiomyocyte	3D tissue model with cells aged by long-term culture	**3D tissue model displayed:** lower survival rate less proliferative capacity higher β-gal activity high ROS lower cardiac beating velocity	Acun et al., 2019
		Null	IPSC --> mDA neurons	Telomere manipulation	**mDA neurons displayed:** DNA damage Increased Mito-ROS loss of mDA neuron marker TH	Vera et al., 2016
		Null	IPSC --> Brain organoid	Organoid exposed to ionizing radiation 0.5 or 2 Gy of 250 MeV protons for 30 mins, 24 h, 48 h	**Cerebral organoid displayed:** Increase in DNA damage	Oyefeso et al., 2023
		Null	IPSC --> Brain organoid	Organoid exposed to hypoxic conditions 0.1 O2 for 24 h	**Cerebral organoid displayed:** BBB dysfunction increased oxidative stress elevated secretion of inflammatory cytokines such as IL-1*β*, TNF*α*, IL-6	Nzou et al., 2020
Werner syndrome-based model	Telomere dysfunction Premature senescence	WRN	WS fibroblasts--> IPSC -- > MSC	Reprogramming by OSKM+HDACi+TGFβ RI kinase I and differentiation with MSC medium containing bFGF	**WS-MSC displayed:** Increase in senescence reduced telomere length higher p53 expression	Cheung et al., 2014
		WRN	WS fibroblasts--> IPSC -- > MSC	hTERT overexpression and p53 knockdown in MSC	**WS-MSC displayed:** Rescues premature senescence Increased telomere length	Cheung et al., 2014 Wang et al., 2018
		WRN	WS fibroblasts--> IPSC -- > MSC	Gene correction of WRN in WS IPSC clones	**WS-MSC displayed:** Higher proliferation Rescues premature senescence Normal karyotype	Kato et al., 2021 Tu et al., 2020
Centenarian-based model	Short telomeres Increased senescence	Null	Supercentenarian (aged 114) B-LCL -- > IPSC -- > MPC	Reprogramming by OSKM	**IPSC clones displayed:** long telomeres normal karyotype	Lee et al., 2020
		Null	Centenarian (aged 106,109) fibroblasts --> IPSC --> neuronal cells	Reprogramming by OSKM	**IPSC clones displayed:** long telomeres higher expression of igf1, igf1r, sirt2, foxo1, and sirt1	Yagi et al., 2012
		Null	Centenarian fibroblasts (aged 92,94,96,101) --> IPSC --> embryonic lineages	Reprogramming by OSKMNL/ inhibition of p53 or p21 CIP1	**IPSC clones displayed:** long telomeres decrease in senescence	Lapasset et al., 2011
Alzheimer’s based model	Accumulation of amyloid-β/tau Increase in p-tau	PSEN1 A246E	FAD IPSC --> NPCs	Reprogramming by OSKM and differentiation by RA and neurosphere formation	**NPCs displayed:** Increase in Aβ 42/40 ratio Increase in premature differentiation and p-tau	Yang et al., 2017
		PSEN1 A246E	FAD IPSC --> NPCs	Reprogramming by OSKM and differentiation by dual SMAD inhibition	**NPCs displayed:** Increase in Aβ 42/40 ratio	Sproul et al., 2014
		PSEN1 V89l	FAD IPSC --> NPCs	Reprogramming by OSKM and differentiation by dual SMAD inhibition + BDNF	**NPCs displayed:** Increased proliferation neurite outgrowth	Pansri et al., 2021
		PSEN1 A246E/ PSEN2 N141l	FAD IPSC --> neurons	Reprogramming by OSKLN and differentiation in hIPSC medium w/o bFGF	**Neurons displayed:** higher secretion of amyloid-β42	Yagi et al., 2011
		PSEN1A246E/H163R/M146L	FAD IPSC --> neurons	Reprogramming by OSKM and differentiation by dual SMAD inhibition	**Neurons displayed:** Increase in Aβ 42/40 ratio	Liu et al., 2014
		PSEN1 A246E	FAD IPSC --> neurons	Reprogramming by OSKM and differentiation by dual SMAD inhibition	**Neurons displayed:** Mitophagy impairment Dysfunctional mitochondria	Martin-Maestro et al., 2017
		APPDp	FAD IPSC --> neurons	Reprogramming by OSKM + EGFP in one third of culture and differentiation by BDNF/GDNF/cAMP	**Neurons displayed:** Increase in amyloid-β40/p-tau/GSK3β	Israel et al., 2012
		APPDp	FAD IPSC --> neurons	Reprogramming by OSKM and differentiation by dual SMAD inhibition	**Neurons displayed:** Increase in Aβ42/40 ratio Increase in p-tau	Moore et al., 2015
		AppDp/PSEN1 M146l, A264E	FAD IPSC --> brain organoid	3D brain organoid (protocol as in Kadoshima et al., 2013)	**Brain organoids displayed:** amyloid plaque deposition hyperphosphorylation of tau endosomal abnormalities	Raja et al., 2016
		PSEN1 A246E/ PSEN2 N141l	FAD IPSC --> brain organoid	3D brain organoid (protocol as in Lancaster et al., 2014)	**Brain organoids displayed:** large Aβ aggregates Increase in Aβ42/40 ratio higher p-tau Developmental defects premature neuronal differentiation	Vanova et al., 2023
		PSEN1 A246E	FAD IPSC --> brain organoid	3D brain organoid (protocol as in Lancaster et al., 2014)	**Brain organoids displayed:** amyloid plaque deposition neurofibrillary tangles	Gonzalez et al., 2018
		Isogenic APOE3, APOE4	APOE4 isogenic IPSC lines --> brain organoid	3D brain organoid (protocol as in Park et al., 2021)		Park et al., 2021
		SAD APOE3/3, APOE4/4	SAD IPSC --> brain organoid	3D brain organoid (protocol as in Lancaster et al., 2014)	**Brain organoids displayed:** amyloid-β accumulation hyperphosphorylation of tau (independent of APOE status)	Hernandez et al., 2021
		53 participants in cohort (16 particpants with clinical and pathological diagnosis of AD)	PBMC-- > IPSC--> induced neurons (iNs)	Reprogramming by sendai virus and differentiation by neurogenin-2 direct induction protocol	Neuritic plaque burden in brain correlated with intra Aβ42/40 ratio in iNs of same individual Tau tangle measures in brain negatively correlated with p-tau in iNs but p-tau levels in brain and iNs were positively correlated. APOE4/4,3/3,2/2 displayed no differences in tau levels	Valentina et al., 2021
Parkinson’s based model	Accumulation of α-synuclein Increased ROS Mitochondrial dysfunction	SNCA triplication	PD IPSC--> mDA neurons	Reprogramming by OSKM and differentiation based on dual inhibition by noggin and SB431542+Dorsomorphin	**DA neurons displayed:** higher levels of α-synuclein	Devine et al., 2011
		SNCA triplication	PD IPSC--> mDA neurons	Reprogramming by OSKM and differentiation protocol as described in Byers et al., 2011	**DA neurons displayed:** higher levels of α-synuclein higher oxidative stress	Byers et al., 2011
		SNCA triplication/ SNCA A53T	PD IPSC--> mDA neurons	Reprogramming by OSKM and differentiation protocol as described in Kriks et al., 2011	**DA neurons displayed:** intracellular accumulation α-synuclein in TH+ cells elevated α-synuclein release	Zambon et al., 2019
		LRRK2 mutant G2019S	PD IPSC-- > DA neurons	Reprogramming by OSK and differentiation protocol as described in Nyugen et al., 2011	**DA neurons displayed:** higher oxidative stress mitochondrial dysfunction impairment in protein degradation	Nguyen et al., 2011
		PARK2/PINK1	PD IPSC-- > DA neurons	Reprogramming by OSKM and differentiation by dual-SMAD inhibition	**DA neurons displayed:** mitochondrial dysfunction Increased oxidative stress elevated α-synuclein synaptic dysfunction DA accumulation	Chung et al., 2016
		PINK1/LRRK2	PD IPSC--> neurons	Reprogramming by OSK and differentiation protocol as described in Cooper et al., 2012	**Neurons displayed:** Increased ROS mitochondrial dysfunction	Cooper et al., 2016
		PINK1	PD IPSC--> mDA neurons	Reprogramming by OSKM and differentiation protocol as described in Chambers et al., 2009	**DA neurons displayed:** impaired mitochondrial recruitment of PINK1	Seibler et al.,2011
		PARK2/PINK1	PD IPSC-- > DA neurons	Reprogramming by OSKM and differentiation protocol as described in Shaltouki et al., 2015	**DA Neurons displayed:** synuclein accumulation mitochondrial dysfunction	Shaltouki et al., 2015
		p.D620N VPS35	PD IPSC-- > DA neurons	Reprogramming by OSKM and differentiation protocol as described in Hanss et al., 2021	**DA Neurons displayed:** synuclein accumulation mitochondrial dysfunction Impaired mitochondrial respiration Increased ROS	Hanss et al., 2021
		p.D620N VPS35	PD IPSC-- > DA neurons	Reprogramming by OSKM and differentiation protocol as described in Doi et al., 2014	**DA Neurons displayed:** synuclein accumulation in TH+ cells Endosomal dysfunction	Bono et al., 2020
		LRRK2 G2019S	PD IPSC-- > 3D organoids	3D organoid protocol as described in Fiore et al., 2022 (Matrigel based)	**DA neurons displayed:** higher levels of α-synuclein	Fiore et al., 2022
		Null	IPSC-derived brain endothelial cells, pericytes, astrocytes, microglia, and dopaminergic neurons--> Brain chip model of substantia nigra (exposed to α-Syn fibrils)	Substantia nigra brain chip model based on organ-on-chips technology to recapitulate synucleopathy of PD	**DA neurons displayed:** accumulation of synuclein impaired mitochondria neuroinflammation compromised BBB function	Pediaditakis et al., 2021

Reprogramming of PSEN1 mutant A246E fibroblasts to IPSCs by conventional OSKM factors and differentiation to NPCs by dual-smad inhibition resulted in the increase in Aβ 42/40 ratio in NPCs compared to the control^86^. Furthermore, when PSEN1 mutant A246E is reprogrammed by the similar reprogramming factors and differentiated by retinoic acid (RA) and neurosphere formation, the resulting neurons displayed increase in Aβ 42/40 ratio, p-tau, and premature differentiation replicating tau pathology effectively^87^. Besides the primary features of AD such as β-amyloid and tau pathology, premature neuronal differentiation is widely reported to be one of early pathological features of AD^88–91^. On the other hand, PSEN1 mutant V89l hNPCs reprogrammed by OSKM reprogramming strategy and differentiated with dual SMAD inhibition, when treated with BDNF (Brain-derived neurotrophic factor), promotes neurite outgrowth and increases cell proliferation in neurons, suggesting that a combination of factors could be more effective in disease recapitulation^92^. This indicates that the reprogramming by typical OSKM factors in addition to the suitable differentiation protocol would be sufficient for recreating β-amyloid, and tau pathology in PSEN mutant NPCs.

IPSC model of FAD-patients carrying APP^Dp^ (amyloid-β-precursor protein, Dp=duplication) were generated from fibroblasts by the addition of OSKM and EGFP in one-third of the culture^93^. Neurons generated from the APP^Dp^ patients through differentiation by BDNF/GDNF/cAMP displayed a 3.5-fold increase in the expression of amyloid-β ^1–40^, a 2-fold increase in p-tau, and a 2-3-fold increase in GSK-3β activity, large early endosomes than the non-demented control (NDC). A similar reprogramming and differentiation protocol employed in one of the SAD patients also resulted in the differentiated neurons exhibiting 3-fold increase in amyloid-β ^1–40^, a 2-fold increase in p-tau, large early endosomes and GSK-3β activity than NDC suggesting the protocol might be effective in recapitulating both amyloid and tau pathology irrespective of mutation background^93^. However, it must be noted that only one of the SAD patient-derived neurons exhibited Alzheimer’s phenotype suggesting a possible limitation. Another study which utilized OSKM for reprogramming followed by differentiation by dual-SMAD inhibition in addition to retinoid successfully recapitulated AD phenotypes such as increase in Aβ peptides and p-tau in cortical neurons derived from FAD- IPSC of APP^Dp^ ^94^.

In comparison to 2D, 3D models harbouring APP^Dp^ or PSEN1 mutant recapitulate AD disease pathology with mature phenotypes^90,95,96^. 3D brain organoid generated from FAD-IPSCs harbouring APP^Dp^ or PSEN1 mutation M146l/A246E displayed amyloid plaque deposition and hyperphosphorylation of tau at 60 days and 90 days of in-vitro culture respectively^95,97^. Additionally, FAD organoids displayed higher levels of small and large endosomes compared to the control organoid, recapitulating endosomal abnormalities of AD. A comparable recapitulation of amyloid plaques and neurofibrillary tangles was seen in the FAD PSEN1 mutant A246E derived cerebral organoid^96,98^. A similar organoid generation protocol^98^ employed in PSEN1 mutant A246E and PSEN2 mutant N141l recapitulated AD phenotypes such as aggregation of Aβ peptides, hyperphosphorylation of tau, increase in Aβ42/40 ratio, premature neuronal differentiation, and developmental and tissue pattern defects^89^.

In relative to FAD, LOAD demonstrates a notable correlation with the Aβ42/37 ratio, likely influenced by the complex interplay of genetic variants^26^. Cerebral organoids developed from APOE4 isogenic IPSC lines exhibited both amyloid-beta (Aβ) deposition and hyperphosphorylation of tau protein, with APOE ε4 demonstrating higher levels of pathogenic proteins compared to APOE ε3^99^. A similar disease recapitulation demonstrated by another study^100^ also suggested that the conversion of APOE4 to APOE3 resulted in the decreased p-tau (ser 199), however variation in overall disease phenotypes were found to be mostly independent of APOE status. This is in line with the observation which states that the APOE 4/4,3/3,2/2 haplotypes displayed no differences in tau levels in the IPSC-derived iNs of AD individuals^26^.

#### Parkinson’s

Parkinson’s disease (PD) is characterized by the progressive degeneration of DA neurons in the substantia nigra compacta caused by the aggregation of α-synuclein in the form of Lewy bodies^101,102^. PD could be hereditary or sporadic (patients generally carry single nucleotide polymorphism). Mutations in the genes of α-synuclein (*SNCA*), leucine-rich repeat kinase 2 (*LRRK2*), PTEN-induced putative kinase 1 (*PINK1*), parkin RBR E3 ubiquitin-protein ligase (*PARK2*), and cytoplasmic protein sorting 35 (*VPS35*) are associated with PD.

Various missense mutations as well as the multiplication of SNCA locus cause misfolding or overproduction of α-synuclein protein, an attribute of PD pathology^103,104^. However, mutations linked to SNCA are relatively rare and often associated with young-onset Parkinson’s. PINK1 plays a role in guiding parkin, an E3 ubiquitin-protein ligase, to the outer mitochondrial membrane of dysfunctional mitochondria promoting mitophagy and removal of damaged mitochondria by ubiquitination. Consequently, mutations in these genes result in impaired mitophagy and thereby accumulation of damaged mitochondria^105,106^. Approximately 5-10% of familial Parkinson’s disease cases are attributed to LRRK2 mutations, such as N1437H, R1441, Y1699C, and G2019S. These mutations have the potential to augment the kinase function of LRRK2 and are associated with the accumulation of the α-synuclein^107^. Recent findings also indicate that LRRK2 can inhibit lysosomal degradation in microglia and macrophages, with this effect being exacerbated by the hyperactivation of LRRK2’s kinase activity. This suggests an elevated risk of Parkinson’s disease due to LRRK2 mutations, stemming from the impairment of the lysosomal degradation^108^. VPS35 is one of the key components of the cargo-binding retromer complex facilitating transport between membrane-bound organelles and plasma membranes^109,110^. Given that VPS35 has established functional interactions with parkin^111,112^ and LRRK2^109,113^ it is plausible that the observed lysosomal and autophagy dysfunction, disrupted mitochondrial function resulting from perturbed mitochondrial homeostasis, and alterations in neurotransmission may be linked to these interactions^114–117^.

#### Strategies for modelling Parkinson’s

Reprogramming fibroblasts from PD patients with SNCA triplication using conventional OSKM factors (Fig. 1) and differentiation by dual inhibition^118^ or differentiation by removal of growth factor^119^ resulted in DA neurons that exhibited approximately twice the levels of α-synuclein compared to unaffected first-degree relatives^67,118,119^. Employing a similar reprogramming and floor plate based differentiation method^120^ in PD patients with SNCA triplication or SNCA A53T mutation showed no significant changes in α-synuclein protein levels in differentiated DA neurons^121,122^. However, the intracellular accumulation of α-synuclein in TH^+^ cells increased by 4-fold, and 3-fold in SNCA triplication and SNCA A53T mutant DA neurons, respectively. This suggests that reprogramming using OSKM factors along with the relevant differentiation protocol^118–120^ might be effective in replicating synuclein pathology in DA neurons.

In addition to SNCA triplication, other PD-patient cells with mutations like LRRK2, PINK1, PARK2, and VPS35 were also successfully reprogrammed by the conventional OSKM/OSK factor and diverse differentiation methods^117,123–128^. Neurons derived from LRRK2 mutant G2019S-IPSCs exhibited greater susceptibility to oxidative stress, mitochondrial dysfunction, reduced neurite length and impairment in the protein degradation^129^. Moreover, IPSC-derived DA neurons carrying PINK1/PARK2 mutations exhibited mitochondrial dysfunction such as decreased mitochondrial respiration, proton leakage, abnormal mitochondrial morphology, and impaired mitochondrial movement, in addition to α-synuclein accumulation, a typical characteristics of PD pathology^124–128,130^. DA neurons carrying VPS35 mutation displayed PD pathologies such as increased mitochondrial ROS, decreased basal and maximal respiration, endosomal dysfunction and α-synuclein accumulation^131,132^.

Apart from 2D, 3D model of PD offers additional advantages like recapitulation of complex and mature PD pathophysiology involving discrete brain cell types, and BBB^133–136^ (Table 1). The tissue-engineered 3D model from hiPSCs-derived DA neurons with the LRRK2 mutant G2019S exhibited elevated levels of α-synuclein (approximately twofold) compared to 2D cultures^134^. Furthermore, 3D midbrain organoid with LRRK2 G2019S mutant (generated by CRISPR-cas9 technology), displayed decreased neurite length, increase in the levels of p-α-syn (7-fold), and increased expression of TXNIP (Thioredoxin Interacting Protein)^137^. TXNIP, an endogenous inhibitor of thioredoxin (TRX) system, is found to increase during the development of AD neurons^138,139^. Additionally, it was found that the amyloid-β peptide (Aβ_1-42_) promotes tau phosphorylation at Ser202/Thr205 through activation of p38 MAPK in SH-SY5Y cells suggesting the critical role of TXNIP in AD pathogenesis.

A more sophisticated PD model requires co-culture of other brain cell types with DA neurons^135,136^. 3D substantia nigra brain-chip model containing IPSC-derived DA neurons, astrocytes, microglia, pericytes, and microvascular brain endothelial cells under fluid flow manifest synucleinopathies by introduction of α-syn fibril into brain channel^135^. Following exposure to α-syn fibril for 6 days, the resulting 3D model displayed increase in the accumulation of p-α-syn (approx. 10-fold), mitochondrial impairment, increased ROS production, activation of astrocytes and microglia, increased levels of secreted cytokines causing neuroinflammation and dysfunction in BBB permeability. These findings suggests that the 3D microenvironment plays a crucial role in the accurate recapitulation of mature disease phenotype in vivo.

#### Different routes of neuronal differentiation

**Fig. 2:**
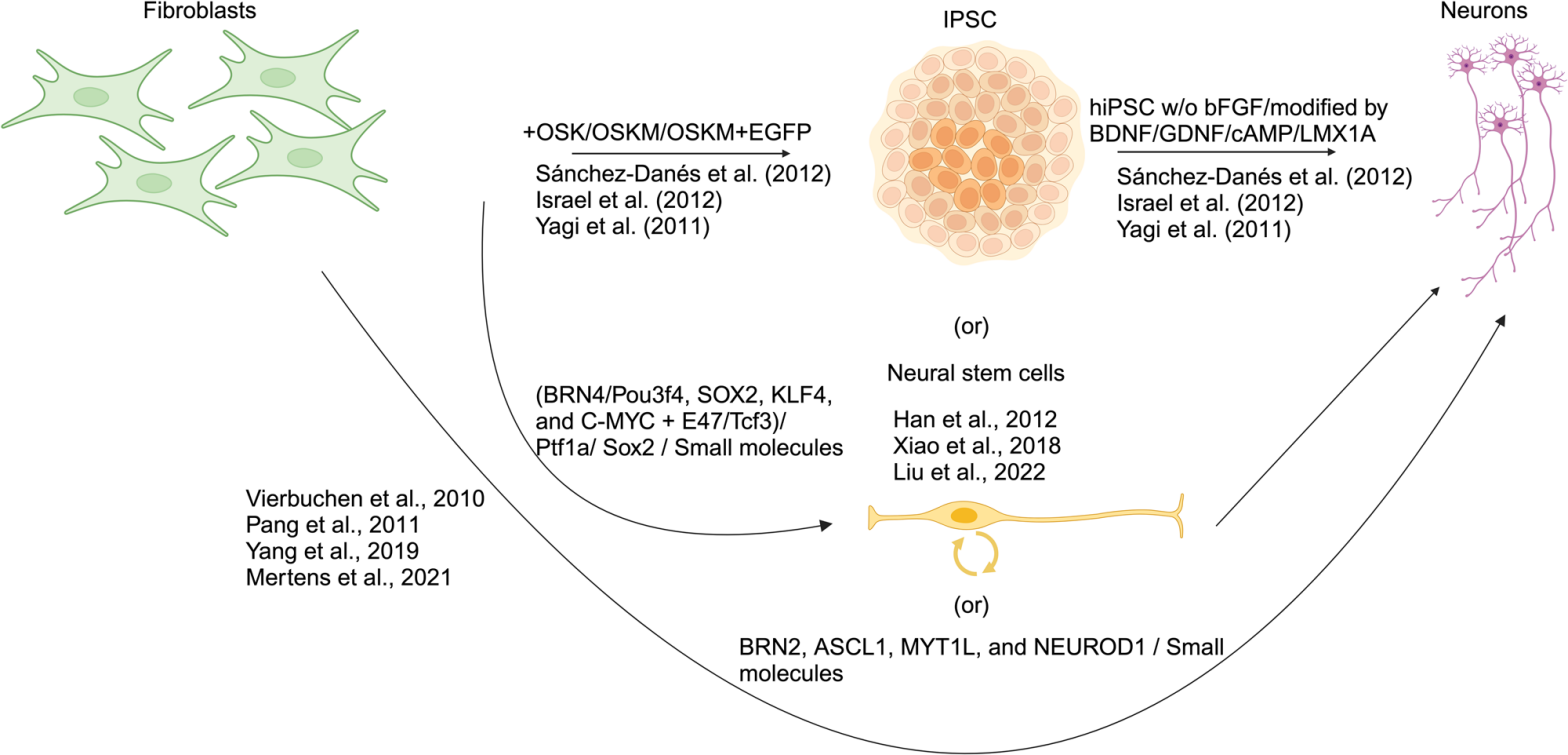
Different routes of neuronal differentiation. Schematic model depicting three ways of neuronal differentiation (generated in Bio-render). 1) Conventional route to reprogram IPSC from fibroblasts followed by subsequent differentiation into neurons. 2) Reprogramming of fibroblasts into neural stem cells (NSCs) followed by differentiation into neurons. 3) Direct transdifferentiation of fibroblasts to neurons.

Multiple strategies were employed to obtain neuronal cells from fibroblasts. The conventional way is to reprogram fibroblasts to IPSCs using Yamanaka factors^67^ and then differentiate them into neurons^67,120,140,141^ (Fig. 2). The major limitation of this approach is that the differentiation of cells from IPSCs can be incomplete and could lead to tumorigenesis^142,143^. In addition, the rejuvenation that occurs during reprogramming can reset the aging characteristics of the donor cells^31^.

On the other hand, fibroblasts can be reprogrammed into neural stem cells (NSCs) and then differentiated into neurons. Transcription factors including BRN4/Pou3f4, SOX2, KLF4, and C-MYC plus E47/Tcf3 can be used to reprogram fibroblasts to induced neural stem cells (iNSCs)^144^ (Fig. 2). The induced neural stem cells, thus generated can self-renew, carry the epigenetic features of the donor, and are highly identical to NSCs. This method overcomes the ethical concerns associated with stem cell isolation from foetal brain tissues and has a promising role in cell replacement therapy (CRT) due to its non-tumorigenic ability. Surprisingly, a group of researchers reported having reprogrammed fibroblasts to NSCs using a single transcription factor like Sox2^145^ or Ptf1a (Pancreas transcription factor 1 subunit alpha)^146^. Out of these two, the reprogramming efficiency of Ptf1a seems to be 0.4% greater than that of Sox2 at day 14. Ptf1a regulates the expression of genes involved in the development and differentiation of specific cell types, in the pancreas and cerebellum. The reprogramming ability of Ptf1a requires the interaction of Ptf1a with Rbpj (a regulator of notch signalling), followed by subsequent activation of transcription factor (TF) genes like Sox, bHLH, homeobox, and POU domain, and Notch signalling components driving neural stem cell (NSC) homeostasis^146^. The resulting iNSCs could differentiate into functional neurons and could significantly improve the cognitive ability of AD mouse models upon transplantation. However, iNSCs generated by these methods can pose safety concerns for clinical application due to the ectopic expression of genes and viral vectors. Recent reports^147^ suggest a safer way of reprogramming by employing small molecules like Bix01294, RG108, CHIR99021, Ascorbic acid, Repsox, LDN193189, Y27632, and Q-VD-OPh. iNSCs thus generated expressed neural markers like SOX2, PAX6, Nestin, Olig2, L1cam, MAPT, CNTN1, MAP2, and NeuN. However, it should be noted that these cells exhibited robust proliferation capabilities, hindering their differentiation. Consequently, a DNA alkylating and cross-linking agent called mitomycin C (MMC), was employed to suppress their proliferation before switching to a neural differentiation medium. Moreover, the utilization of different cell states for reprogramming resulted in varying levels of reprogramming efficiency.

An efficient approach is to directly reprogram fibroblasts to neurons^148–150^. This offers an advantage as the inducible neuronal cells generated by this approach carry genetic and epigenetic marks of the patients and it skips the need for IPSC generation and the potential risk for tumorigenesis. This method makes use of transcription factors (TFs) like BRN2, ASCL1, MYT1L, and NeuroD1 (Fig. 2) with a reprogramming efficiency of 19.5% with Mouse embryonic fibroblasts (MEFs) as a starting cell. The iN cells produced by this protocol displayed neuronal traits, such as the expression of neuronal markers like MAP2 and synapsin, as well as the ability to form functional synapses and generate mature action potential. It was observed that ASCL1 alone is likely capable of inducing certain neuronal characteristics, such as the generation of action potentials, but the co-infection of additional factors aids in the process of neuronal maturation. Direct differentiation can also be achieved rapidly in 10 days by introducing a cocktail of small molecules such as CHIR99021 (GSK-3 inhibitor), LDN193189 (TGFβ inhibitor), SB431542 (ALK5 inhibitor), RG108 (DNA methyltransferase inhibitor), dorsomorphin (AMPK inhibitor), forskolin (Adenylyl cyclase activator), Y27632 (ROCK1 inhibitor), DAPT (γ-secretase inhibitor), purmorphamine (Hedgehog agonist), ISX9 (Neurogenic Modulator), and P7C3-A20 (Neurogenic Modulator)^151^. Furthermore, the addition of A83-01 (inhibitor of ALK signalling) or PD0325901 (Mitogen-activated kinase inhibitor) increased the reprogramming efficiency by 15%. Nonetheless, this approach is not advantageous to direct the generation of specific neurons as transcription factors play a decisive role in directing the development of specific neuron subtypes over others. Therefore, this protocol could prove advantageous for rapid and safer reprogramming without the use of viral vectors provided there is no requirement for producing a particular subtype of neuron. However, a combination of transcription factor (Ngn2/Ascl1 based) and cocktail of small molecules can be employed for the direct conversion of fibroblast to specific subtype of neuronal conversion. The neuronal conversion was achieved in 3 weeks and the cultures majorly consisted of glutamatergic neurons with a minor fraction of GABA^+^ neurons^150^.

On the other hand, physical factors like radio-electric asymmetric conveyer (REAC) can also be employed to directly reprogram fibroblasts into neurons^152^. REAC delivering radio-electric fields at the frequency of 2.4 GHz induces the transcription of Oct4, Sox2, cMyc, Nanog, and Klf4 in hSF (Human skin-derived fibroblasts) at 6-20 h after treatment followed by a decline during differentiation. hSFs upon REAC treatment for 72 h facilitate cardiac differentiation by a steady increase in the expression of Nox4, reaching the maximum at 7-10 days. Moreover, REAC treatment in ES (Embryonic Stem) cells significantly increased the expression of GATA4, neurogenin1, and myoD essential for cardiogenesis, neurogenesis, and myogenesis^153^. The main advantage of this method is that the need to use unsafe viral-mediated gene delivery is eliminated.

#### Clinical application

The ability of the IPSCs to differentiate into various brain cells has made it possible to establish 3D and co-culture models, enabling a deeper understanding of the mechanism of brain-related disorders. Neurons derived from the AD patient’s IPSC harbouring specific mutations manifest distinct AD-associated phenotypes. This makes it possible to investigate the impact of mutation-associated phenotypes present in AD patients^93,154^. For instance, IPSC-derived excitatory neurons lacking CLU (clusterin) were less sensitive to Aβ- induced toxicity^155^. Moreover, the generation of isogenic APP and PSEN1 mutant IPSCs using CRISPR/Cas9 knock in has revealed a shared early endosomal enlargement phenotype among APP and PSEN1 mutant neurons^156^. These observations confirms that the neurons derived from IPSC models retain the disease-associated genetic phenotype.

Furthermore, the 3D co-culture developed can offer valuable insights into the effect of AD-linked mutation on the interaction of neuronal-neuronal or neuronal-non-neuronal disease. Moreover, 3D co-culture involving multiple brain types can serve as a promising target for drug development. Although no absolute barrier exists in deriving neuronal or non-neuronal cells from IPSC, the ability to derive mature IPSC-derived brain cells is a major challenge. 3D co-culture can bypass such limitations by facilitating maturation through cell-cell interaction. In addition, direct differentiation of fibroblasts to neurons mimics the disease-related phenotypes more accurately by retaining age-related cellular, transcriptomic, and epigenetic alterations after differentiation.

Additionally, IPSC-derived neuronal cells can be used as a platform for drug screening. Treatment of IPSC-derived neuronal cells with GSI (γ-secretase inhibitor), NSAID (non-steroidal anti-inflammatory drug), and BSI (β-secretase inhibitor) resulted in the decreased Aβ production^157^. Notably, the efficacy of GSI on γ-secretase was found to be higher in the late differentiation stage at day 52, likely due to lower levels of γ-secretase components in the early differentiation stage at day 38, influencing the sensitivity of γ-secretase to GSI. This suggests that the late differentiated neuronal cells are a pre-requisite for drug screening. Another study evaluated the effectiveness of the clinically disapproved drug DHA (Docosahexaenoic acid) on IPSC-derived neurons from AD patients carrying APP-E693, APP-V717L, and sporadic AD mutant (AD8K213). The effectiveness of DHA was found to be correlated with the intracellular accumulation of Aβ oligomers, explaining the differential drug responsiveness of DHA in only a subset of AD patients in clinical trials^158^. Moreover, IPSC-based screening of an array of pharmaceutical compounds revealed that a combination of drugs can improve the Aβ phenotype of AD^159^. Anti-Aβ cocktails consisting of bromocriptine, cromolyn, and topiramate decreased toxic Aβ levels by 40% and 20-30% in IPSC-derived neurons of familial and sporadic AD patients, respectively. Genome-wide association studies (GWAS) of IPSC-derived cortical neurons of 102 AD patients revealed that 24 genome wide loci were linked with Aβ pathology^160^. Notably, CTNNA3 and ANO3 were primarily associated with Aβ 42/40 ratio, while a rare variant of KCNMA1 were linked to the amount of Aβ 42, suggesting Aβ pathology in AD is mainly influenced by polygenicity of neurons.

Besides the progress of research in neurodegenerative diseases, the IPSCs can be potentially used to treat AD and PD patients. For example, IPSCs transplanted in AD mice and differentiated into glial cells resulted in a decrease of aggregated Aβ and an improvement in cognitive abilities^161^. In PD mice and primates, transplanted IPSC-derived neurons led to better motor function^162,163^. First clinical studies in humans showed an increase in dopamine uptake as well as an improvement in the quality of life^164^. Hence, the generation of patient-derived disease models will not only offer insights into aging and age-related diseases but also have a significant role in clinical application.

## Conclusion

For better treatments and a deeper understanding of aging and age-related diseases, the strategies discussed in this review provide a valuable tool for researchers. By addressing the limitations in the reprogramming and differentiation strategies, we can improvise the utilization of iPSCs as a powerful tool for advancing our understanding of aging/age-related diseases.
